# Twelve-Month Contraceptive Supply Policies and Medicaid Contraceptive Dispensing

**DOI:** 10.1001/jamahealthforum.2024.2755

**Published:** 2024-08-30

**Authors:** Maria I. Rodriguez, Thomas H. A. Meath, Ashley Daly, Kelsey Watson, K. John McConnell, Hyunjee Kim

**Affiliations:** 1 Center for Reproductive Health Equity, Department of Obstetrics and Gynecology, Oregon Health & Science University, Portland; 2 Center for Health Systems Effectiveness, Oregon Health & Science University, Portland

## Abstract

**Importance:**

Nineteen states have passed legislation requiring insurers to cover the dispensation of a 12-month supply of short-acting, hormonal contraception.

**Objective:**

To determine whether 12-month contraceptive supply policies were associated with an increase in the receipt of 12-month or longer supply of contraception.

**Design, Setting, and Participants:**

This retrospective cohort study included data from all female Medicaid enrollees aged 18 to 44 years who used short-acting hormonal contraception (ie, pill, patch, or ring) from 2016 to 2020.

**Exposures:**

Eleven treatment states where legislation required insurers to cover a 12-month supply of contraception to continuing users and 25 comparison states without such legislation prior to December 2020.

**Main Outcomes and Measures:**

Proportion of contraception months received via a single 12-month or longer fill.

**Results:**

This study included 48 255 512 months of dispensed oral pill, patch, and ring contraception prescription supply among 4 778 264 female Medicaid enrollees. The majority of months of supplied contraception were for the contraceptive pill rather than the patch or ring. In a staggered difference-in-differences model, the 12-month supply policy was associated with an estimated 4.39–percentage point (pp) increase (95% CI, 4.38 pp-4.40 pp) in the proportion of contraception dispensed as part of a 12-month or longer supply, from a mean of 0.11% in treatment states during the first quarter of the study period. Investigating the heterogeneity in policy association across states, California stood out with a 7.17-pp increase (95% CI, 7.15 pp-7.19 pp) in the proportion of contraception dispensed as a 12-month or longer supply; in the other 10 treatment states, the policy association was less than 1 pp.

**Conclusions and Relevance:**

In this cohort study of Medicaid recipients using short-acting hormonal contraception, the passage of a 12-month contraceptive supply policy was associated with a minimal increase in the proportion of contraception dispensed through a 12-month or longer supply.

## Key Points

**Question:**

How was legislation in 11 states requiring insurers to cover 12 months of short-acting hormonal contraception associated with the receipt of a 12-month or longer contraceptive supply?

**Findings:**

This national cohort study of Medicaid enrollees found that legislation mandating insurance coverage of a 12-month supply was associated with a modest 4.39–percentage point increase in the proportion of contraception months received via a single 12-month or longer fill. California accounted for the majority of the policy effect with a 7.17–percentage point increase; in other treatment states, it was less than 1%.

**Meaning:**

Medicaid policies requiring coverage of a 12-month supply have not led to substantial increases nationally in prescriptions covering 12-month or longer contraceptive supply, leaving many Medicaid enrollees at increased risk for unintended pregnancy.

## Teaser

This cohort study aims to determine whether 12-month contraceptive supply policies were associated with an increase in the receipt of 12-month or longer supply of contraception.

## Introduction

The ability to decide if or when to become pregnant is fundamental to individual rights, health, and people’s role in society.^1^ Contraception is a safe and highly effective intervention to prevent pregnancy. In the US, the oral contraceptive pill remains the most common form of reversible contraceptive used.^2^ With perfect use, it has 99% efficacy in preventing pregnancy, but its effectiveness is lower with typical use.^3^ A common cause for the decreased effectiveness observed with the pill is breaks in contraceptive use. Common reasons for breaks in contraceptive use are running out of a prescription or a lapse in obtaining a refill.^3^ Evidence supports that dispensing a 6- to 12-month supply of short-acting contraceptive methods (ie, pill, patch, or ring) is associated with improved contraceptive continuation, fewer breaks in coverage, and health system savings.^4,5,6,7^ Despite these findings, research has shown that 70% of short-acting contraceptive users in the US receive a contraceptive supply of 3 months or less.^8^

To address this barrier to contraceptive use, policymakers have enacted 12-month contraceptive supply policies in 19 states. These policies require insurers to cover the cost of dispensing a full 12 months of coverage at once per prescription.^9^ In the absence of such policies, clinicians can prescribe a 12-month supply, but insurance coverage typically dictates the amount a person receives.^10^ For these policies to be effective, however, insurance companies must comply and be held accountable for following the revised coverage guidelines. Similarly, clinicians would need to change their standard prescribing patterns to write for an extended supply of contraception, and pharmacists would need to dispense the full supply.

Limited data have demonstrated whether these policies have been fully implemented and led to changes in prescribing practices. Data from 1 state demonstrated an increase in the overall mean supply of contraceptives dispensed but did not find an association between the policy change and receipt of a 12-month supply.^11^ Whether these results are generalizable to the 18 other states implementing similar policies is not known. This national cohort study assessed the association of 12-month contraceptive supply policies with receipt of a 12-month or longer supply among a national population of Medicaid enrollees.

## Methods

### Study Design and Population

The data in this cohort study consisted of national Medicaid claims and enrollment data covering the years 2016 to 2020, obtained from the Transformed Medicaid Statistical Information Systems (T-MSIS) Analytic Files (TAF). The study’s dataset enabled us to capture each Medicaid enrollee’s demographic information and receipt of contraception. We followed the Strengthening the Reporting of Observational Studies in Epidemiology (STROBE) reporting guideline. The institutional review board at Oregon Health & Science University approved the study protocol. Informed consent was not required because the data were deidentified.

This cohort study included both treatment and comparison states. Treatment states were those with Medicaid legislation or guidance that mandated the coverage of a 12-month supply of contraception, with compliance dates falling between January 1, 2016, and December 31, 2020. We divided this time into 2 periods: (1) the prepolicy period, from January 1, 2016, to the compliance date, and (2) the postpolicy period, from the compliance date to December 31, 2020. Comparison states were those without a policy compliance date prior to December 31, 2020. We identified states with 12-month supply of contraception policies and their compliance date using a 2022 report from Power to Decide.^9^

We excluded 3 states (Washington, Oregon, and South Carolina), as they had already implemented a 12-month supply of contraception prior to the start of the study period. Additionally, 8 states (Arkansas, Florida, Maine, Minnesota, Mississippi, North Carolina, Ohio, and Rhode Island) were excluded due to high concern or unusable TAF data quality based on the CMS Data Quality Atlas.^12^ Furthermore, 2 states (Colorado and Wisconsin) were excluded because they had greater than 10% invalid quantity or days-supply values in prescriptions. Finally, 2 states (Kansas and Nebraska) were removed due to unstable time trends for the receipt of a 12-month or longer supply of contraception that could not be attributed to any specific policy change (eAppendix 1 in Supplement 1).

Of the remaining 36 states, 11 states enacted a 12-month contraceptive supply policy during the study period across 7 waves: (1) quarter 4 of 2016 (Vermont); (2) quarter 1 of 2017 (California, District of Columbia, Hawaii); (3) quarter 3 of 2017 (New York); (4) quarter 1 of 2018 (Nevada); (5) quarter 3 of 2018 (Delaware, Massachusetts, Maryland); (6) quarter 1 of 2019 (New Hampshire); and (7) quarter 1 of 2020 (New Mexico). The study’s comparison group consisted of 25 states that had not implemented a 12-month contraceptive supply policy before the end of 2020 (Alaska, Alabama, Arizona, Connecticut, Georgia, Iowa, Idaho, Illinois, Indiana, Kentucky, Louisiana, Michigan, Missouri, Montana, North Dakota, New Jersey, Oklahoma, Pennsylvania, South Dakota, Tennessee, Texas, Utah, Virginia, West Virginia, and Wyoming).

These data included all short-acting contraceptive prescriptions to Medicaid-enrolled women aged 18 to 44 years in the 11 treatment and 25 comparison states from January 1, 2016, to December 31, 2020. We did not apply restrictions based on enrollment criteria. We excluded prescriptions for individuals with restricted benefits due to citizenship (eg, Emergency Medicaid). We identified contraceptive prescriptions based on a modified version of the Office of Population Affairs’ Contraceptive Care metric. This validated metric uses diagnosis, procedural, and National Drug Codes to capture and classify contraceptive methods.^13^ Lastly, we excluded prescriptions that were missing both days’ supply and National Drug Code quantity dispensed, as well as those with days’ supply that were not multiples of standard contraception pack sizes and those that were unreasonably long (>450 days). For each prescription, we abstracted information on the enrollee’s age, type of short-acting contraception used (pill, patch, and ring), and months of supply received. We aggregated this prescription-level data to the state-quarter level.

### Main Outcomes

The primary outcome was the proportion of total months of contraception supplied during a state-quarter, provided in a single 12-month or longer supply fill. We calculated the length of contraception supply for each prescription fill using information about the days-supply dispensed. When days-supply dispensed was not available, we calculated the length of supply using the quantity of the National Drug Code prescribed on the claim, accounting for standard pack sizes for contraceptives (eAppendix 2 in Supplement 1).

The secondary outcome was the proportion of contraceptive months dispensed as part of a 2- to 3-month supply fill, calculated in the same manner as the primary outcome described previously.

### Statistical Analysis

We used a staggered difference-in-differences model, as described in Sun and Abraham,^14^ to assess the association between the implementation of a 12-month contraceptive supply policy and the study outcomes. This approach allowed us to account for variations in policy implementation timing and estimate the association of the policy on outcomes for the entire sample and across treatment states over time.

The analysis was conducted at the state-quarter level. We used multivariable linear regression models with interaction terms between binary variables for the policy treatment state and binary variables for each quarter relative to policy implementation. States with later implementation dates have more preperiod indicators and fewer postperiod indicators, with each state having between 3 and 16 of each, totaling 19 combined quarter indicators as main explanatory variables. The quarter directly prior to policy implementation served as the reference quarter. The combined group of all states that did not implement the policy served as a comparison group. The models also adjusted for state fixed effects to account for time invariant differences between states, and quarter fixed effects to account for secular time trends in outcomes. State-quarter observations in the model were weighted by the total months supplied to account for differences in population size and contraception supply in each state-quarter.

We adjusted for the mean age of Medicaid enrollees associated with each prescription fill during the quarter in each state. We did not account for race or ethnicity due to unreliable information in TAF regarding these demographics. We did not adjust for health conditions or other patient-level characteristics, as these factors were not expected to affect the amount of contraception supply prescribed. After estimating the model, we averaged coefficients for the quarter by state interaction terms to produce average policy effects: averaging across all postpolicy terms to produce the policy estimates for the entire population, averaging across the postpolicy terms for each state to produce state-level estimates, and averaging across the terms for each state to produce quarter-level estimates. Coefficients were weighted based on the total months of supplied contraception for states and quarters included in the coefficient when estimating average policy effects. We tested for parallel prepolicy trends between each treatment state and comparison states as a proxy for whether changes over time would be similar for the treatment and comparison groups in the absence of the policy (one of the main assumptions of the difference-in-differences model; eAppendix 4 in Supplement 1). All statistical tests were 2-sided, and statistical significance was set at *P* < .05. We used R statistical software, version 4.2.3 (R Project for Statistical Computing), for all analyses and data management.

## Results

**Figure 1.**
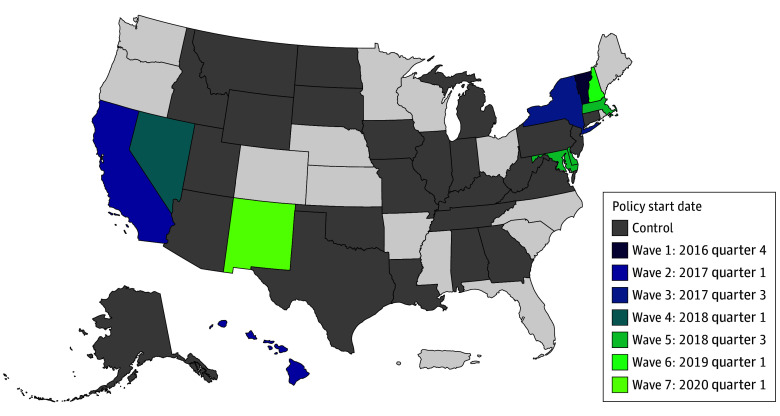
Map of 12-Month Supply Medicaid Policy Implementation Waves and Comparison States

This cohort study included 48 255 512 months of oral pill, patch, and ring contraception prescription fills prescribed to 4 778 264 female Medicaid enrollees aged 18 to 44 years from 2016 to 2020 (eAppendix 3 in Supplement 1). These prescriptions were for enrollees living in either 11 treatment states where a 12-month contraceptive supply policy was implemented during the study period or 25 comparison states. Treatment states were primarily located in the western or northeastern regions of the US (Figure 1).

The predominant contraceptive type across both treatment and comparison states was oral contraceptive pills (Table 1). In comparison states, the majority of months of contraception were received through just a 1-month supply in both the first (82.73%) and final quarter (51.40%) of the study period (Table 2). In treatment states, 58.10% of months of contraception were provided through a 1-month supply in the first quarter of the study, which dropped to 31.23% by the final quarter. This was accompanied by an increase in the receipt of a 2- to 3-month supply of 19.36 percentage points (pp) in treatment states and 31.34 pp in comparison states. Notably, contraception months provided as part of a 12-month or longer supply was virtually nonexistent in comparison states (0.01% in the first quarter and 0.02% in the final quarter) during the study period. In treatment states, such prescriptions were only 0.11% in the first quarter of the study but increased to 6.16% by the final quarter (eAppendix 6 in Supplement 1).

**Table 1.** Short-Acting Hormonal Contraception by 12-Month Policy, 2016-2020

Characteristic	Group of states (N = 48 255 512 mo)	
	Treatment	Comparison
Months of contraception, No.	24 066 051	24 189 461
Patient age, mean (SD), y	28.0 (6.8)	26.8 (6.8)
Method, %		
Pill	87.8	90.1
Patch	5.5	4.3
Ring	6.8	5.6

**Table 2.** Monthly Contraception Supply Proportion by Policy, 2016-2020

Dispensed contraception supply, mo	Group of states (N = 5 193 995 mo), %					
	Treatment			Comparison		
	Study start^a^	Study end^b^	Raw difference	Study start^a^	Study end^b^	Raw difference
1	58.1	31.2	−26.9	82.7	51.5	−31.2
2-3	41.5	60.9	19.4	17.1	48.4	31.3
4-5	0.2	0.7	0.6	0.0	0.0	0.0
6-7	0.1	0.8	0.7	0.1	0.0	−0.1
8-11	0.0	0.2	0.2	0.0	0.0	0.0
≥12	0.1	6.2	6.1	0.0	0.0	0.0

^a^ Study start shows raw outcome rates for the first quarter of the study period (January 1, 2016, to March 31, 2016).

^b^ Study end shows raw outcome rates for the last quarter of the study period (October 1, 2020, to December 31, 2020).

**Figure 2.**
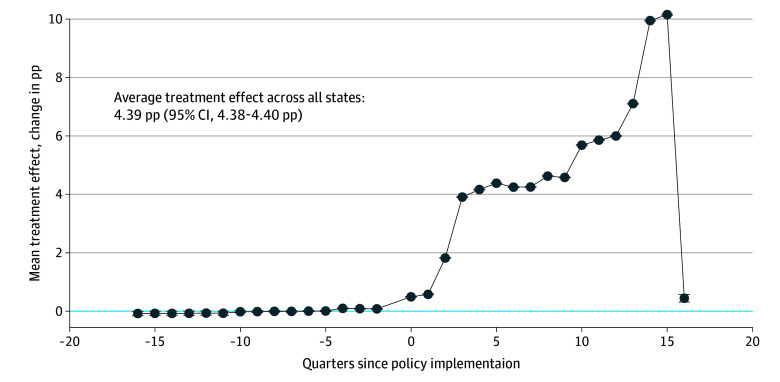
Estimated Average Treatment Effects of 12-Month Contraception Fills on Medicaid Enrollees by Quarter The average impact of 12-month contraception fills on the proportion of contraception months received by Medicaid enrollees is shown, broken down by quarter, both before and after policy implementation. The horizontal error bars indicate 95% CIs. The estimated average treatment effect is measured in percentage points (pp).

The association of the policy with the proportion of contraception months dispensed via a single 12-month or longer supply in each state for each postpolicy quarter was determined using a staggered difference-in-differences analysis (Figure 2). On average, the policy implementation was associated with a 4.39-pp increase (95% CI, 4.38 pp-4.40 pp) in the proportion of contraception received from a single 12-month or longer supply fill, from a mean of 0.11% in treatment states during the first quarter of the study period. Figure 2 drops sharply in the final quarter. This is because each data point represents the average treatment effect for that quarter, among states contributing data. In the final quarter, only Vermont had sufficient data to contribute, and thus the average treatment effect is much lower than in quarters 1 through 15.

**Figure 3.**
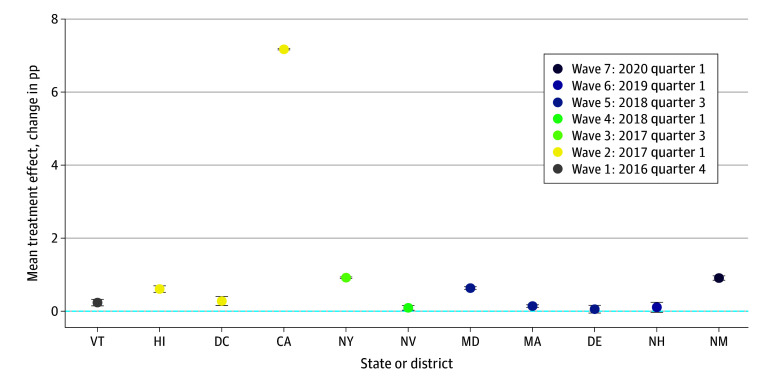
Estimated Average Treatment Effects of 12-Month Contraception Fills on Medicaid Enrollees by State The average treatment effect of 12-month contraception fills on the proportion of contraception months received by Medicaid enrollees is illustrated across different states. The error bars indicate the 95% CIs. The estimated average treatment effect is measured in percentage points (pp).

To further explore these findings, the average policy effects were estimated for each treatment state in the sample, averaging over all postpolicy periods (Figure 3). Notably, California stood out with a 7.17-pp increase (95% CI, 7.15 pp-7.19 pp) in the proportion of contraception months dispensed as a 12-month or longer supply, while the policy effect was less than a 1-pp increase in all other 10 treatment states. This suggests that the primary policy effect presented earlier (4.40-pp increase) across all states was predominantly driven by a relatively larger policy effect observed in California.

Tests of parallel preintervention trends showed qualitatively similar results for the prepolicy time trends for treatment states, as compared to the trend in the comparison states (eAppendix 5 in Supplement 1). There was a small but significant relative downward time trend for California. This difference in trends was very small (less than −0.1 pp per quarter) and would be expected to bias the positive policy effect estimates downward toward a null result. There was also a small but significant relative upward time trend for New Mexico (less than 0.01 pp per quarter) and would be expected to bias the positive policy effect estimate upwards.

No statistically significant evidence suggested the policy was associated with a change in the proportion of contraception dispensed as part of a 2- to 3-month supply fill (eAppendix 7 in Supplement 1). Tests of parallel preintervention trends did not show statistically significant evidence of trend differences for the 2- to 3-month supply outcome.

## Discussion

In this retrospective national cohort study, we found that passage of a policy requiring insurers to cover dispensation of a 12-month supply was associated with only a small increase (4.40 pp) in months of contraception received through a 12-month or longer supply among Medicaid enrollees. This suggests that 12-month contraceptive supply policies have not been fully implemented, with only a small number (6.06%) of contraceptive months received from a 12-month prescription in the final quarter of the study.

Importantly, in the first quarter of the study, the majority of contraception received by Medicaid enrollees was through 1-month supply fills (82.73% in comparison states and 58.10% in treatment states). This is an outdated and potentially harmful approach to contraceptive care: dispensing only 1 month of pills at a time is associated with decreased continuation of contraception and increased unintended pregnancies.^5,15^ Although we did observe a shift by the final quarter of the study in 1-month prescriptions, these accounted for a smaller proportion of overall months supplied (a decrease of 26.88% in treatment states and 31.23% in comparison states). This is an inadequate response, particularly in light of decreased access to safe abortion services in the US.^16^

Multiple factors may explain this essentially null finding. For 12-month contraceptive supply policies to have an impact, contraceptive users need to be aware of their rights, clinicians need to change their prescribing patterns, pharmacists must agree to dispense the full amount, and insurance companies must comply with coverage. The findings of this study suggest that implementation has not fully occurred. We restricted the study sample to the Medicaid population to eliminate uncertainty in insurance coverage: Medicaid programs are subject to and compliant with state laws. A prior study from Oregon identified a small increase in mean supply dispensed following a 12-month policy change, mainly within Medicaid-enrolled women and Title X clinics.^11^ Previous research has demonstrated that the majority of clinicians were unaware of these laws and had not adjusted prescribing patterns accordingly.^17,18^ A national survey of obstetrics and gynecology trainees indicated that fewer than 5% were regularly writing prescriptions for a 12-month supply.^18^ Similarly, a qualitative study of pharmacists in Massachusetts demonstrated limited awareness of the law.^19^ These are key barriers to policy implementation.

Notably, California did have a higher increase in contraception received via a 12-month supply fill following the policy, but this was still only a small increase (7.17%). California’s long-standing and robust publicly funded family planning network may have helped to increase the messaging to clinicians to change prescribing habits.

### Limitations

This cohort study was limited in that it relied on administrative data, and we did not have information on individual fertility preferences. We also lacked information on additional demographic and clinical variables that may have impacted contraceptive use, such as pregnancy history. Some individuals might not want a 12-month or longer supply; for example, if they wanted to become pregnant within the next year. The use of claims data allowed us to see what was reimbursed by the insurer, but we were unable to see the quantity that was initially prescribed and compare this to what was dispensed by the pharmacist or reimbursed by the insurer. We were unable to account for whether prescriptions were new, or how much time was left before the prescription would expire. This may have affected the amount dispensed in some intervention states. A few states initially restricted new prescriptions to a 3-month fill or limited the months that can be dispensed if a prescription is close to its expiration dates. The study period included the early phase of the COVID-19 pandemic when disruptions in health care were common. However, previous research has demonstrated that the early phases of the COVID-19 pandemic were not associated with a change in contraceptive supply dispensed.^20^

## Conclusions

We found that policies mandating a 12-month contraceptive supply coverage in 11 states were associated with a small increase in the proportion of contraception supply dispensed in quantities covering a 12-month or longer period. Medicaid policy has failed to be fully implemented in the states where these policies were passed. Full implementation of these policies requires outreach to contraceptive users, prescribers, pharmacists, and payers, as well as enforcement from state governments. Federal policy mandating coverage of a 12-month supply is another strategy to support access for individuals using short-acting contraception.

## Notes

Supplement 1. eAppendix 1. State selection eAppendix 2. Outcome eAppendix 3. Study cohort creation eAppendix 4. Staggered difference-in-differences model specification eAppendix 5. Tests of parallel preintervention trends eTable. Differences in prepolicy trends of 12 month contraceptive supply by state eAppendix 6. Trends in months of contraception received as part of a 12-month supply over time eAppendix 7. Estimated average treatment effect on the likelihood of receiving a 2- to 3-month contraceptive supply eReferences

Supplement 2. Data Sharing Statement
