# Supplementary material for: Twelve-Month Contraceptive Supply Policies and Medicaid Contraceptive Dispensing
Source: JAMA Health Forum. 2024 Aug 30;5(8):e242755. doi: 10.1001/jamahealthforum.2024.2755 (PMC11364992; doi:10.1001/jamahealthforum.2024.2755)
Supplement: Supplement 1. — eAppendix 1. State selection eAppendix 2. Outcome eAppendix 3. Study cohort creation eAppendix 4. Staggered difference-in-differences model specification eAppendix 5. Tests of parallel preintervention trends eTable. Differences in prepolicy trends of 12 month contraceptive supply by state eAppendix 6. Trends in months of contraception received as part of a 12-month supply over time eAppendix 7. Estimated average treatment effect on the likelihood of receiving a 2- to 3-month contraceptive supply eReferences [file jamahealthforum-e242755-s001.pdf]

Supplemental Online Content

Rodriguez MI, Meath THA, Daly A, Watson K, McConnell KJ, Kim H. Twelve-month contraceptive supply policies and medicaid contraceptive dispensing. *JAMA Health Forum.* 2024;5(8):e242755. doi:10.1001/jamahealthforum.2024.2755

eAppendix 1. State selection

eAppendix 2. Outcome

eAppendix 3. Study cohort creation

eAppendix 4. Staggered difference-in-differences model specification

eAppendix 5. Tests of parallel preintervention trends

eTable. Differences in prepolicy trends of 12 month contraceptive supply by state

eAppendix 6. Trends in months of contraception received as part of a 12-month supply over time

eAppendix 7. Estimated average treatment effect on the likelihood of receiving a 2- to 3-month contraceptive supply

eReferences

This supplemental material has been provided by the authors to give readers additional information about their work.

© 2024 Rodriguez MI et al. *JAMA Health Forum*.

Appendix: Examining the association between 12-month contraceptive supply policies and quantity of contraceptives dispensed to Medicaid recipients in the United States

Appendix Section 1. State selection

We used assessments published by the Centers for Medicare & Medicaid Services DQ Atlas, supplemented by our own analyses of data quality, to select states with adequate data quality for analysis. We began with all 50 states and the District of Columbia and looked at data quality assessments for TAF RIF Release 2 from 2016-2018 and Release 1 from 2019-2020. Four states were excluded due to high concern or unusable TAF data quality based on the CMS Data Quality Atlas (DQ Atlas) **[1]**.

- 2 states with high concern Total Medicaid and CHIP Enrollment (Maine and Rhode Island)
- 2 states with high concern National Drug Code in the Rx file (Florida and Arkansas)
- 3 states with high concern Rx claims volume (Minnesota, North Carolina, Ohio)
- 1 state with unusable Rx claims volume (Mississippi)

After calculation of length of supply, we compared values to known lengths of each type of contraception. Oral pill and ring prescriptions were assumed accurate if they were written for a multiple of 21, 28, 30, or 91 days. Patch prescriptions were assumed to be accurate if they were written for a multiple of 7 days. When quantity or days-supply dispensed was not a multiple of a known length it was considered to be invalid. States with >10% invalid prescriptions were excluded from our analysis.

© 2024 Rodriguez MI et al. *JAMA Health Forum*.

- 2 states had >10% invalid quantity or days-supply values in their contraception prescriptions (Colorado and Wisconsin)

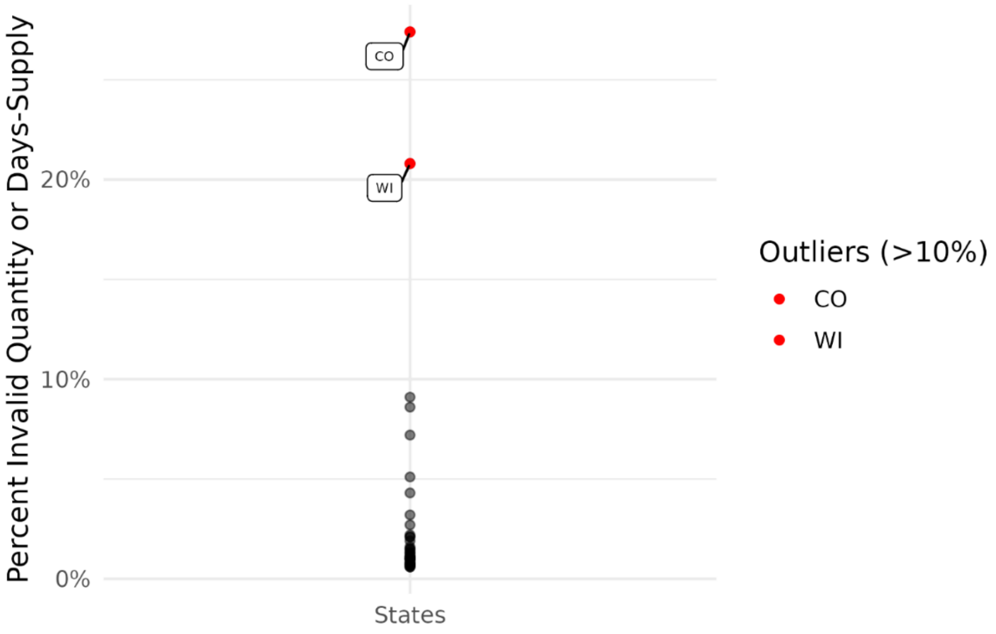

In addition to reviewing length of supply for validity, we assessed fluctuations in the mean length of contraception supply that could not be explained by changes in state policies. These fluctuations could be due to unmeasured policies or poor data quality and were reviewed by a clinician for clinical relevancy.

- 1 state was excluded due to a sudden drop in the mean length of supply at the end of 2016 (Nebraska)
- 1 state was excluded due to a sudden increase in the mean length of supply in the second quarter of 2016 (Kansas)

© 2024 Rodriguez MI et al. *JAMA Health Forum*.

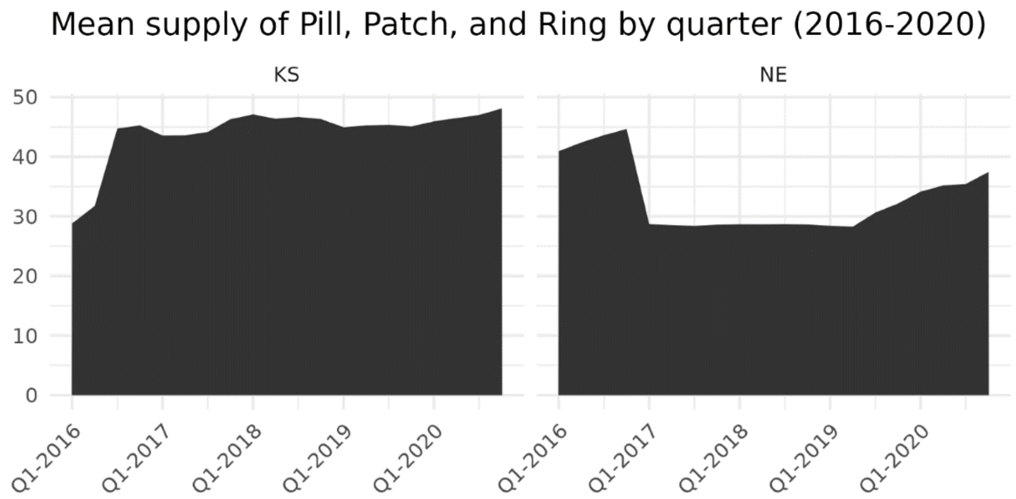

Finally, we excluded states that had implemented 12-month supply prior to the start of the study period.

- 3 states had implemented policies prior to January 1, 2016 (Washington, Oregon, and South Carolina)

© 2024 Rodriguez MI et al. *JAMA Health Forum*.

Appendix Section 2. Outcome

Our primary outcome was the proportion of total months of contraception supplied during a state quarter, provided in single 12-month or longer supply fills, and our secondary outcome was the proportion of contraceptive months dispensed as part of a two to three-month supply.

We identified receipt of contraception using the Office of Population Affair's (OPA) Contraceptive Provision Measure (v3.01). This validated measure uses diagnosis, procedural, and drug codes to capture and classify contraceptive methods. We adapted OPA's Statistical Analysis System (SAS®) program for the calculation of this measure to be run in R software and our unique data environment. Though the ultimate goal of the measure is to identify if a woman was provided any most or moderately effective method of contraception, a contraception claims code set is also available as part of the OPA technical documentation and oral pill, patch, and ring prescriptions are flagged as an intermediary for identifying most effective contraception in their SAS program [2]. We further modified OPA's code to retain these flags and create a dataset of oral pill, patch, and ring prescriptions.

Once we had identified contraception prescriptions we calculated the length of supply. Where available, we prioritized the days-supply dispensed as our measure of length of supply. For oral pill and ring prescriptions, we allowed 21, 28, or 30 days dispensed to represent 1 month. For patches, we allowed 28 days dispensed to represent 1 month, assuming 1 patch is the equivalent of a 7-day supply.

When days-supply dispensed was not available, we imputed the length of supply using the quantity of the NDC being prescribed on the claim, accounting for standard contraception pack sizes.

We made the following assumptions regarding length of supply based on quantities dispensed:

- 1 Oral Pill Pack or 1 Ring Unit = 30 days supply
- 1 Patch = 7 days supply

Prescriptions were flagged as being a 12-month supply of contraception if they had a length of supply between 273 and 450 days.

© 2024 Rodriguez MI et al. *JAMA Health Forum*.

Appendix Section 3. Study cohort creation

Individuals were required to meet the following criteria to be included in this study:

- Women ages 18 to 44 as of December 31 of the measurement year
- Enrolled in Medicaid in one of the 11 treatment and 25 comparison states
    - We did not apply restrictions based on enrollment criteria
- Did not have restricted benefits due to citizenship (e.g. Emergency Medicaid)
- Had a valid oral pill, patch, and ring contraception in the measurement year

Consort Table of Unique Individuals by State

Description	Change	Unique Individuals by State
Start		108,387,283
Restrict to women	-50,897,900	57,489,383
Between the ages of 18 and 44	-32,310,632	25,178,751
Drop records with changing age or sex	-154	25,178,597
Drop those without contraception in the year	-20,005,719	5,172,878
Exclude those with restricted benefits due to citizenship	-62,423	5,110,455
Exclude claims with unknown days supply & quantity	-149	5,110,306
Drop those with duplicate prescriptions	-5,253	5,105,053
Exclude claims with small days supply or quantity	-265,473	4,839,580
Exclude claims with >15 months supply	-6,641	4,832,939
Exclude claims that don't match pack size multiples	-18,126	4,814,813

Unique Individuals (deduplicated across states): 4,778,264

© 2024 Rodriguez MI et al. *JAMA Health Forum*.

Oral pill, patch, and ring contraception prescriptions were required to meet the following criteria to be included in this study:

- Could not be missing both days' supply and National Drug Class (NDC) quantity dispensed
- Must be unique prescriptions
    - Prescriptions were considered to be unique if they contained a unique combination of the following variables: Member ID, Service Date, and NDC
- Must meet minimum quantity and length requirements:
    - 21 days or quantity >=21 for oral contraception
    - 7 days or quantity >= 1 for patch contraception
    - 21 days or quantity >= 1 for ring contraception
- Must be <450 days (or 15 months) in length
- Must be a multiple of standard contraception pack sizes (see Appendix Section 2 for additional details)

Consort Table of Oral Pill, Patch, and Ring Contraception Prescriptions for Women who Met the Study Eligibility Criteria

Description	Change	Prescriptions
Start		36,597,760
Exclude claims with unknown days supply & quantity	-801	36,596,959
Drop those with duplicate prescriptions	-113,092	36,483,867
Exclude claims with small days supply or quantity	-659,523	35,824,344
Exclude claims with >15 months supply	-39,148	35,785,196
Exclude claims that don't match pack size multiples	-268,801	35,516,395

© 2024 Rodriguez MI et al. *JAMA Health Forum*.

Appendix Section 4. Staggered Difference-in-differences model specification

We assessed the association of the policy with dispensing 12 or more months supply using a staggered difference-in-differences model as described in Sun and Abraham, 2021[3]. We modeled our outcome using a linear probability model with the following form:

$$Y_{st} = \beta_0 + \vec{\beta}_1\overrightarrow{State_s} + \vec{\beta}_2\overrightarrow{Quarter_t} + \vec{\beta}_3\overrightarrow{PolicyState_s}\overrightarrow{QuartersFromPolicy_{st}} + \vec{\beta}_4\vec{X}_{st} + \varepsilon_{st}$$

Where Y_{st} represents the outcome Y for state s and quarter t, β_0 is the intercept, $\vec{\beta}_1\overrightarrow{State_s}$ is a vector of state fixed effects to account for baseline differences between states, and $\vec{\beta}_2\overrightarrow{Quarter_t}$ is a vector of quarter fixed effects to account for nationwide secular time trends. Our coefficients of interest are $\vec{\beta}_3$, representing the policy effect estimates associated with each policy state in each month pre- and post-implementation. $\overrightarrow{PolicyState_s}\overrightarrow{QuartersFromPolicy_{st}}$ is a vector of interaction terms between an indicator for the state that implemented the policy (with all non-policy states as the reference group) and the quarters since the policy implementation in that state. These interaction terms are only created for quarters that have study data, and we exclude terms for the quarter prior to the policy implementation as a reference group to avoid overparameterizing the model. For example, Vermont implemented the policy three quarters after the start of the study period, and thus we will create interaction terms for three quarters through two quarters prior to study and for quarters 0 through 16 following the implementation of the policy. In total, the model contains 209 such interaction terms. $\vec{\beta}_4\vec{X}_{st}$ controls for the effect of time-varying state characteristics $\vec{\beta}_4\vec{X}_{st}$, and ε_{st} represents the normally distributed error term.

© 2024 Rodriguez MI et al. *JAMA Health Forum*.

Aggregate measures of treatment effect at the population level, the quarter-level, and the policy implementation state level were estimated by averaging the interaction term coefficients weighted by the relative total months of supplied contraception captured by each quarter and wave combination.

© 2024 Rodriguez MI et al. *JAMA Health Forum*.

Appendix Section 5. Tests of parallel pre-intervention trends

One of the assumptions of the staggered difference-in-differences model is that changes over time in each policy wave would follow the same trend as changes over time in the control wave in the absence of treatment. We tested this assumption by estimating time trends for each state in the pre-policy period using a linear model with interaction terms between policy state and continuous quarters since the start of the study. These interaction terms allowed us to test whether those trends significantly differed from the time trend for the control group of states that did not receive the policy. Estimates for the difference in time trends from the control group for each wave and each outcome are presented below:

Differences in pre-policy trends of the proportion of contraception months received via a 12 month or greater supply fill by state

State	Estimate (percentage points per quarter)	Std. Error	p-value
Vermont	-0.0098	0.0327	0.764
DC	-0.0574	0.0299	0.056
California	-0.0532	0.0035	<0.001
Hawaii	0.0010	0.0206	0.021
New York	-0.0007	0.0026	0.781
Nevada	0.0001	0.0053	0.985
Delaware	0.0003	0.0060	0.966
Massachusetts	0.0005	0.0019	0.797
Maryland	0.0005	0.0023	0.832
New Hampshire	0.0004	0.0059	0.946
New Mexico	0.0047	0.0016	0.0038

© 2024 Rodriguez MI et al. *JAMA Health Forum*.

Appendix Section 6. Trends in months of contraception received as part of a 12-month supply over time

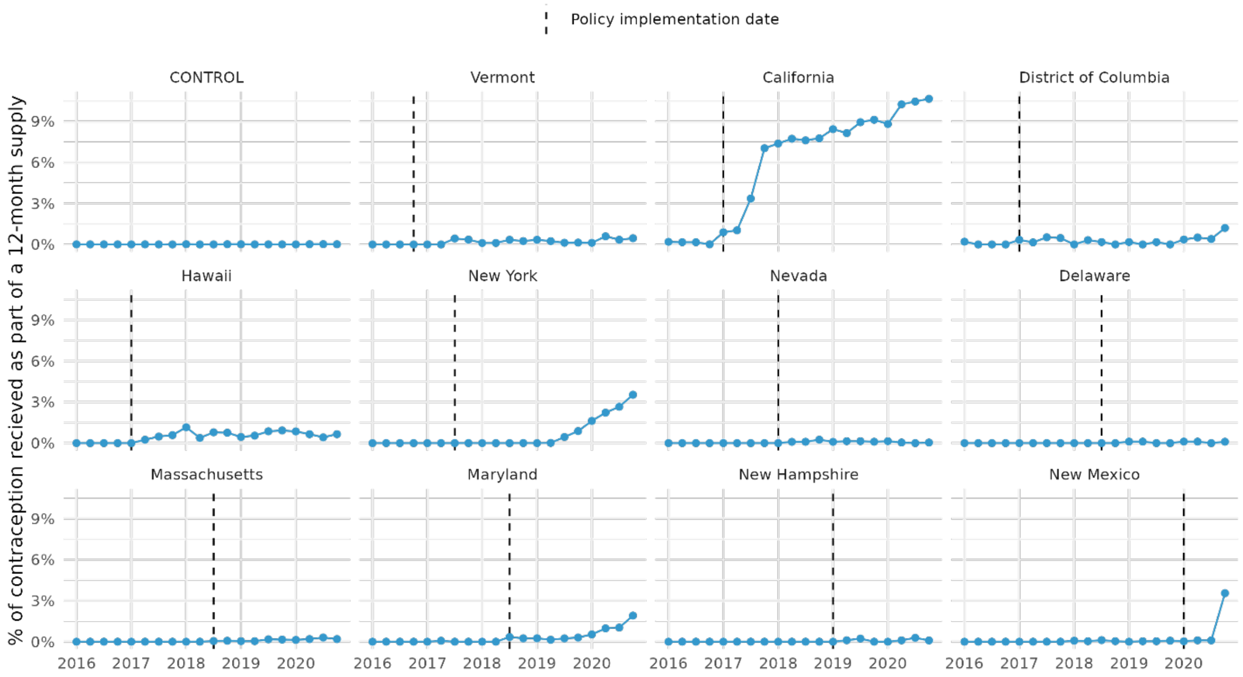

© 2024 Rodriguez MI et al. *JAMA Health Forum*.

Appendix Section 7. Estimated average treatment effect on the likelihood of receiving a 2 to 3-month contraceptive supply

We examined the policy effect on the proportion of contraception months supplied via a single fill of 2 to 3-month supply in each state for each post-policy quarter using a staggered difference-in-differences analysis. On average, the policy implementation was associated with a 0.235% percentage point decrease (95% CI: -7.919, 7.441) in the proportion of contraception received from a single 2 to 3-month or longer supply fill. In addition, we estimated policy effects for each state in the sample, averaging over all post-policy periods. We did not find statistically significant evidence suggesting the policy was associated with a change in the proportion of contraception dispensed as part of a 2 to 3-month supply fill.

© 2024 Rodriguez MI et al. *JAMA Health Forum*.

Estimated average treatment effect on the likelihood of receiving a 2 to 3-month contraceptive supply among Medicaid enrollees in the United States by quarter pre- and post-policy implementation (n= 48,255,512; 2016-2020)

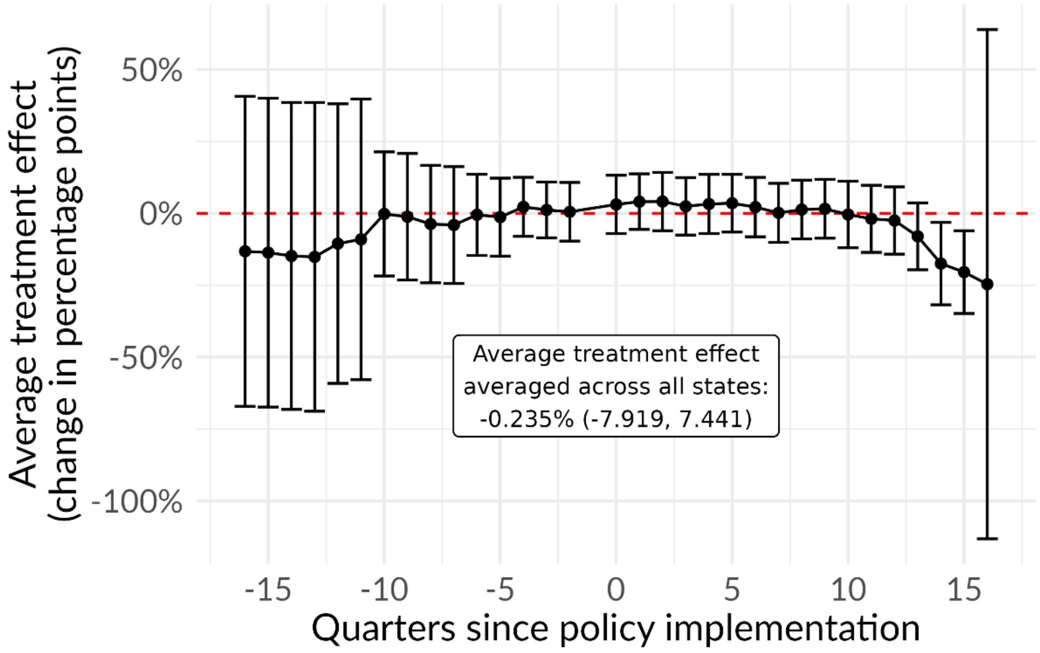

© 2024 Rodriguez MI et al. *JAMA Health Forum*.

Estimated average treatment effect on likelihood of receiving a 2 to 3-month contraceptive supply among Medicaid enrollees in the United States by state (n= 48,255,512; 2016-2020

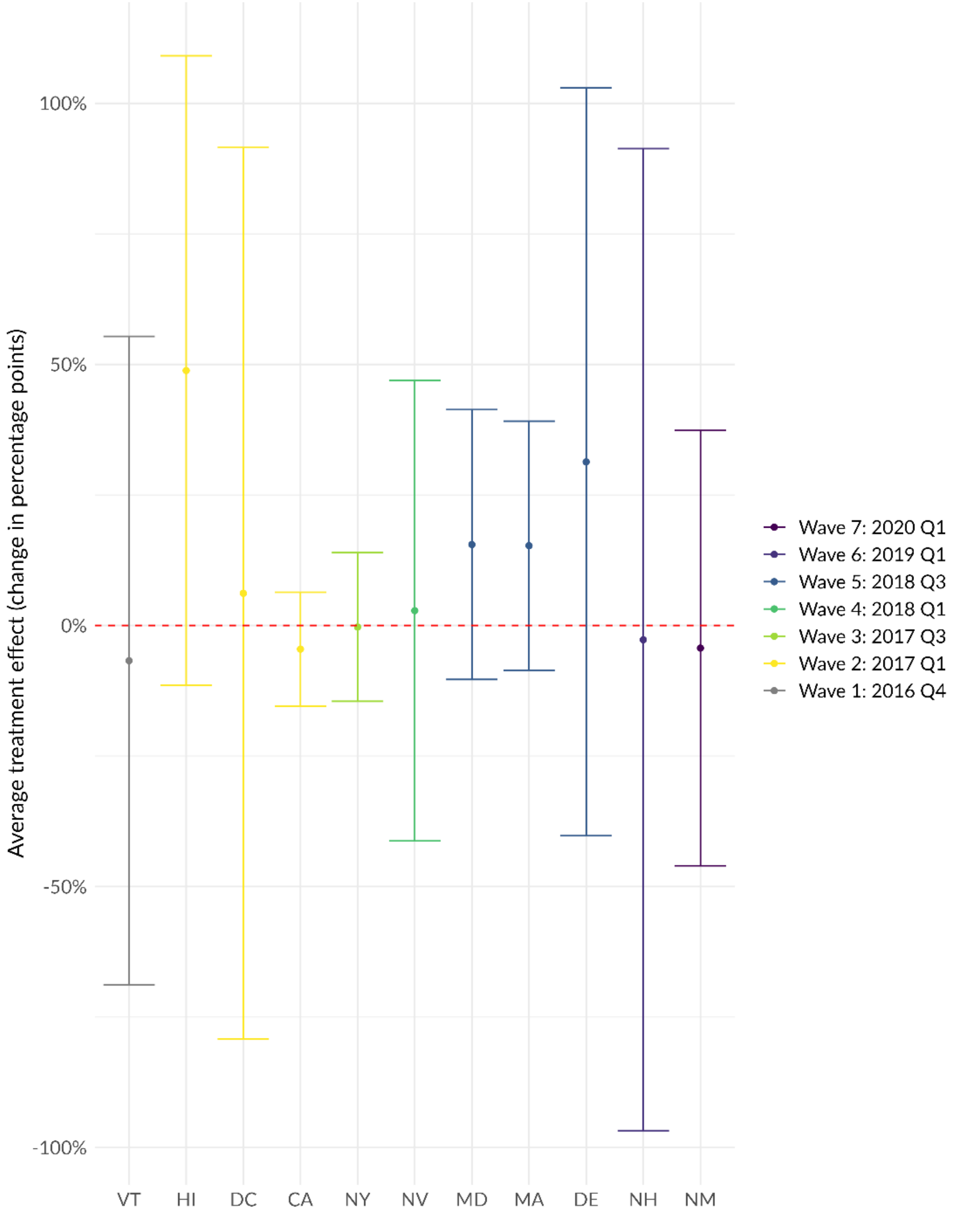

)

© 2024 Rodriguez MI et al. *JAMA Health Forum*.

Works Cited

[1] Centers for Medicare and Medicaid Services. *DQ Atlas*. Accessed March 5, 2024. https://www.medicaid.gov/dq-atlas/

[2] Office of Population Affairs. Contraceptive Provision Measures. Accessed March 5, 2024. https://opa.hhs.gov/claims-data-sas-program-instructions

[3] Abraham S SL. Estimating Dynamic Treatment Effects in Event Studies with Heterogeneous Treatment Effects. Journal of Econometrics. 2021;225.

© 2024 Rodriguez MI et al. *JAMA Health Forum*.
