# Supplementary material for: Twelve-Month Contraceptive Supply Policies and Medicaid Contraceptive Dispensing
Source: JAMA Health Forum. 2024 Aug 30;5(8):e242755. doi: 10.1001/jamahealthforum.2024.2755 (PMC11364992; doi:10.1001/jamahealthforum.2024.2755)
Supplement: Supplement 2. — Data Sharing Statement [file jamahealthforum-e242755-s002.pdf]

Data Sharing Statement

Rodriguez. Twelve-Month Contraceptive Supply Policies and Medicaid Contraceptive Dispensing. *JAMA Health Forum*. Published August 30, 2024. doi:10.1001/jamahealthforum.2024.2755

Data
Data available: No
